# Effectiveness of the Euvichol® oral cholera vaccine at 2 years: A case-control and bias-indicator study in Haiti

**DOI:** 10.1016/j.ijid.2023.11.022

**Published:** 2024-02

**Authors:** Wilfredo R. Matias, Yodeline Guillaume, Gertrude Cene Augustin, Kenia Vissieres, Ralph Ternier, Damien M. Slater, Jason B. Harris, Molly F. Franke, Louise C. Ivers

**Affiliations:** 1 Division of Infectious Diseases, Massachusetts General Hospital, Boston, USA; 2 Division of Infectious Diseases, Brigham and Women's Hospital, Boston, USA; 3 Center for Global Health, Massachusetts General Hospital, Boston, USA; 4 Zanmi Lasante, Croix-des-Bouquets, Haiti; 5 Department of Pediatrics, Harvard Medical School, Boston, USA; 6 Department of Global Health and Social Medicine, Harvard Medical School, Boston, USA; 7 Harvard Global Health Institute, Cambridge, USA

**Keywords:** Euvichol**®**, Cholera vaccine, OCV, Vaccine effectiveness, Haiti

## Abstract

**Objectives:**

The World Health Organization recommends the use of oral cholera vaccine (OCV) in cholera control efforts. Euvichol®, pre-qualified in 2015, is the leading component of the Global OCV stockpile, but data on its field effectiveness are limited. To evaluate Euvichol® vaccine effectiveness (VE), we conducted a case-control study between September 2018 to March 2020 following an OCV campaign in November 2017 in Haiti.

**Methods:**

Cases were individuals with acute watery diarrhea. Stool samples were tested by culture and real-time polymerase chain reaction of the *Vibrio cholerae* ctxA gene. Cases were matched to four community controls without diarrhea by residence, enrollment time, age, and gender, and interviewed for sociodemographics, risk factors, and self-reported vaccination. Cholera cases were analyzed by conditional logistic regression in the VE study. Non-cholera diarrhea cases were analyzed in a bias-indicator study.

**Results:**

We enrolled 15 cholera cases matched to 60 controls, and 63 non-cholera diarrhea cases matched to 249 controls. In the VE analysis, eight (53%) cases reported vaccination with any number of doses compared to 43 (72%) controls. Adjusted two-dose OCV VE was 69% (95% CI –71 to 94%).

**Conclusions:**

Between 10-27 months after vaccination, Euvichol® was effective and similar to Shanchol™, suggesting that it can serve as one component of multi-sectoral comprehensive cholera control.

## Highlights

• The World Health Organization recommends the use of oral cholera vaccines in cholera control efforts.
• We conducted a case-control study of the cholera vaccine, Euvichol® in Haiti.
• Two-dose Euvichol® vaccine effectiveness was 69%.
• Euvichol® can serve as one component of comprehensive cholera control efforts.

## Background

Cholera accounts for a significant burden of morbidity and mortality, with recent years demonstrating an increase in the number of outbreaks globally [1]. As part of comprehensive cholera control efforts, the World Health Organization (WHO) recommends the use of oral cholera vaccine (OCV) in endemic and epidemic settings [2]. A global stockpile of WHO pre-qualified OCVs was established in 2013 and has since supplied the majority of OCVs used in outbreak settings [3].

There are currently three available pre-qualified OCVs: Dukoral® (Crucell, Sweden), a *Vibrio cholerae* O1 whole-cell killed vaccine with addition of recombinant cholera toxin B subunit, Shanchol™ (Shantha Biotechnics-Sanofi Pasteur, India), and Euvichol® (EuBiologics, Republic of Korea), both *V. cholerae* O1 (Inaba and Ogawa) and O139 whole-cell killed vaccines based on a similar formulation, without cholera toxin B subunit [2]. Given their low cost and ease of delivery, Shanchol™ and Euvichol® have been predominantly used for mass vaccination campaigns in global outbreak settings. Euvichol® was prequalified in 2015 and replaced in 2017 by an improved form named Euvichol-Plus® which is contained in a plastic (instead of glass) package, thereby reducing the vial's volume and weight, and facilitating delivery in resource-limited settings [4]. Of the three WHO-prequalified OCVs, only Euvichol-Plus® is now readily available, and Shantha Biotechnics, the producer of Shanchol™, has announced an end to production of this OCV [5]. Several studies of Shanchol™ have established the field effectiveness of OCV in preventing cholera [6]. However, although immunogenicity studies have shown that Euvichol® is as immunogenic as Shanchol™, data on its field effectiveness are limited [7,8].

In Haiti, where cholera has now resurged after its introduction in 2010, OCV campaigns have been implemented as part of cholera control efforts [9]. In 2016, Euvichol® was first deployed in southern Haiti following the impact of Hurricane Matthew on the region [10]. Between November-December, 2017, a campaign using remaining doses of Euvichol® was implemented in the Central Plateau of Haiti. Following the OCV campaign in the Central Plateau, we conducted a case-control study and bias-indicator study to evaluate the field effectiveness of Euvichol®.

## Methods

### Study design, participants, and enrollment criteria

This Euvichol® campaign targeted the estimated 98,564 individuals in the commune of Mirebalais, Haiti. The first dose was distributed between November 15, 2017, and November 21, 2017, and the second dose between December 10, 2017, and December 16, 2017. The estimated two-dose coverage was 71% [11]. This study was a two-part, matched OCV effectiveness case-control study and bias-indicator case-control study [12]. Individuals presenting to the main cholera treatment center in the area of the vaccination campaign were invited to enroll.

Both cases and controls had to have lived in the vaccine catchment area at the time of the vaccine campaign and had to be considered eligible for vaccination at the time of the campaign. Individuals from the communal section of Sarazin were excluded from our analysis since this communal section had already received vaccination with Shanchol™ in 2014 in addition to the Euvichol® campaign in 2017, which may have affected effectiveness [13]. Our current study included individuals who lived in Mirebalais for at least one night in the week before presentation to the study site, and sought medical treatment for acute non-bloody diarrhea (defined as three or more loose, watery or liquid stools in a 24-hour period with an onset of 3 days or fewer before presentation) [14], and for whom the diarrheal episode was confirmed as cholera. The bias-indicator study examined the relationship between vaccination and non-cholera diarrhea. It included individuals for whom the diarrheal episode was negative for *V.cholerae* but otherwise met the same criteria as the OCV effectiveness study.

In both studies, each case was matched to four community controls by neighborhood (i.e., localité (French), an administrative division in Haiti), calendar time (i.e., controls were enrolled as soon as possible and at most, within 2 weeks of case enrollment), age, and gender when possible. Controls were considered eligible if they did not have diarrhea, did not seek medical treatment for diarrhea between the date that study enrollment began and the corresponding case's symptom onset, and lived in the same neighborhood as a corresponding case at the time of study initiation. Controls reporting that they would never seek medical care for severe sickness or diarrhea were excluded.

### Procedures

Trained study staff implemented study protocols in their native Haitian Creole using handheld mobile devices. For cases of acute watery diarrhea presenting to the study site, clinical data were abstracted from clinical charts. A study interview was then conducted using a survey tool that determined sociodemographic characteristics, and self-reported individual and household risk factors for cholera such as water, sanitation, and hygiene practices, and known cholera exposure history. Study staff then visited the case's home to conduct a household observation survey. Following enrollment of cases, study staff visited the case's community to identify and enroll community controls using the same survey tools. Vaccination status, including the number of doses received, was assessed by self-report during study interviews. For those who reported vaccination, study staff asked to see a vaccination card. When a vaccination card was not available, to improve the validity of self-reported vaccination status, study staff would ask clarifying questions that are unique to OCV in the region, such as whether the vaccine was received during a mass vaccination campaign on a given date, whether the vaccine was taken orally (as opposed to via injection) and whether the vaccine came in a bottle. They also used visual aids and showed participants images of the vaccine itself.

Stool samples from cases with diarrhea were collected for culture that was performed in Haiti by degree-holding laboratory technicians trained in stool culture methods. Additionally, stool samples were spotted and dried on filter paper and shipped to Massachusetts General Hospital, Boston, MA, USA, where they were screened for *V. cholerae* using a TaqMan Real-Time polymerase chain reaction (RT-PCR) to the *ctxA* gene (Supplementary Materials).

Stool culture and RT-PCR were used to detect the presence of *V. cholerae*. PCR methods were used in this study and are increasingly employed in cholera vaccine effectiveness (VE) studies given that they are more sensitive than traditional culture methods, including in Haiti [15], [16], [17], [18], [19]. A case of cholera diarrhea was an individual with a positive RT-PCR result for *V. cholerae*. A case of non-cholera diarrhea was an individual with a negative RT-PCR. As a supplementary sensitivity analysis, VE using a culture-based definition of cholera was also calculated.

### Statistical analysis

Incident cholera (for the cholera case-control study) and non-cholera diarrhea (for the bias-indicator study) vs control status were the primary outcomes. The primary exposure of interest was the self-reported receipt of two doses of OCV, the recommended Euvichol® immunization schedule. Self-reported vaccination status was used instead of using vaccination cards to confirm vaccination because the availability of vaccination cards was low. We also compared self-reported receipt of one dose to no doses received. We used conditional logistic regression adjusted for potential residual confounding by matching factors (gender and age, modeled as a continuous variable) to calculate odds ratios, 95% CIs, and *P*-values. Because the annual incidence of cholera was low in Haiti over the time of the study, odds ratios were expected to approximate risk ratios. Known risk factors for cholera were identified *a priori* as potential confounders*.* The small number of cholera cases precluded adjustment for all these potential confounders; therefore, we identified the most likely confounders based on the data. We used univariable conditional logistic regression adjusted for matching factors to test the association between these risk factors and cholera, and univariable logistic regression to test the association between these risk factors and vaccination. Variables associated with cholera and vaccination at a significance level of *P* < 0.20 in univariable analyses were included in multivariable models of VE. We calculated VE using the formula: VE = (1 - relative risk) [20]. All analyses were conducted using the *survival* package in R V4.2.2 [21].

### Ethical considerations

We obtained written informed consent from all participants, or a health-care proxy if the participant was unable to consent. For children younger than 18 years of age, we obtained consent from a parent or guardian. We obtained assent from children aged 7-17 years. The study protocol was approved by the Mass General Brigham Human Research Committee (Protocol MGB # 2018P000350) and the Zanmi Lasante Institutional Review Board (Protocol ZL IRB # 113).

### Role of the funding source

Funding sources had no role in the design and implementation of the study, data collection, analysis, interpretation, or in the writing and publication of study results.

## Results

**Figure 1:**
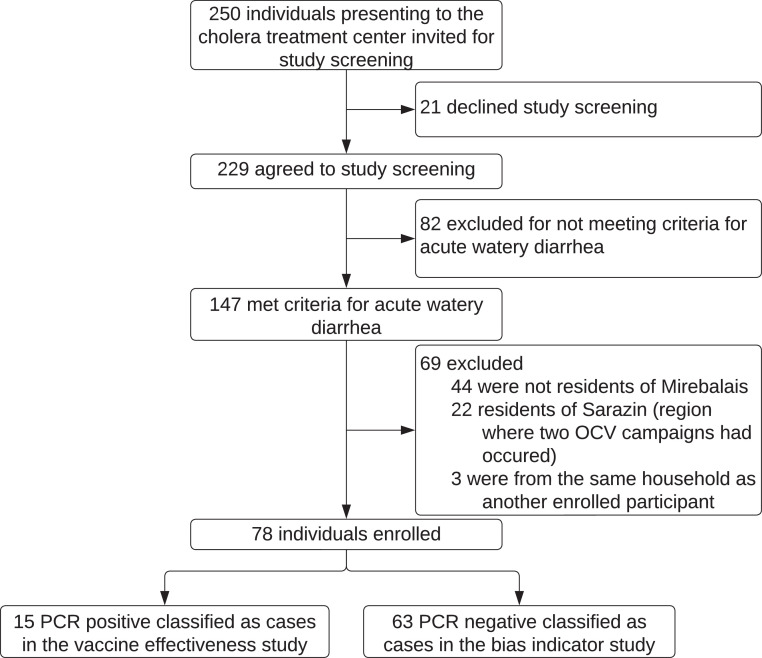
Flow diagram of study participants. OCV, oral cholera vaccine; PCR, polymerase chain reaction.

We enrolled individuals from September 12, 2018, to March 12, 2020. Enrollment was stopped in March 2020 because no culture-confirmed cases of cholera had been detected nationally for nearly 1 year at that time. Over the study period, 250 individuals presented to the cholera treatment center and were screened for participation in the study. 78 individuals met all eligibility criteria and were enrolled in the study. Eight individuals were RT-PCR positive and culture positive for *V. cholerae* (all serotype Inaba) (Supplementary Table 1). Seven individuals were RT-PCR positive, culture negative. Sixty-two individuals were RT-PCR negative, culture negative. One individual was RT-PCR negative but missing culture results. No individuals were RT-PCR negative, culture positive. Given the superior sensitivity of PCR over culture for patients with a clinical syndrome of cholera, all 15 individuals who were RT-PCR positive were included as cases of cholera [22,23] (Figure 1). The median RT-PCR cycle threshold for cases of cholera was 22.0 (19.6-24.7). The 63 individuals with diarrhea who were negative for cholera by RT-PCR were enrolled as cases in the bias-indicator study (Figure 1). Compared to cases of non-cholera diarrhea, cases of cholera were more likely to present with severe dehydration, require intravenous fluids, and be hospitalized overnight (Supplementary Table 1). Sixty controls were matched to the 15 cases of cholera, and 249 controls were matched to cases of non-cholera diarrhea. Characteristics of cases and controls are shown in Table 1.

**Table 1:** Characteristics of cholera cases, non-cholera diarrhea cases, and controls in a case-control study in Haiti, 2018-2020^a^.

	Cholera vaccine effectiveness case-control study			Bias-indicator case-control study		
	Cholera diarrhea cases (n = 15)	Controls (n = 60)	*P*-value^b^	Non-cholera diarrhea cases (n = 63)	Controls (n = 249)	*P*-value^b^
Gender (women) (%)	7 (46.7)	28 (46.7)		31 (49.2)	142 (57.0)	
Age (years)	43.00 [30.00, 85.00]	42.00 [22.00, 54.00]		24.00 [5.50, 45.00]	25.00 [6.00, 44.00]	
Agriculture as income-generating activity	8 (53.3)	46 (76.7)	0.027	26 (41.3)	95 (38.2)	0.732
Attended any school	3 (20.0)	28 (46.7)	0.063	35 (55.6)	163 (65.5)	0.136
Household size	4.00 [3.00, 6.00]	4.00 [3.50, 5.50]	0.565	4.00 [3.00, 6.00]	5.00 [4.00, 6.00]	0.148
Likelihood of poverty	83.60 [42.45, 94.00]	62.70 [40.10, 94.00]	0.259	44.80 [27.70, 80.05]	62.70 [27.70, 83.60]	0.017
Household hunger scale						
Little to no hunger in the household	5 (33.3)	38 (63.3)	Ref	45 (71.4)	211 (84.7)	Ref
Moderate hunger in the household	8 (53.3)	21 (35.0)	0.053	15 (23.8)	36 (14.5)	0.032
Severe hunger in the household	2 (13.3)	1 (1.7)	0.019	3 (4.8)	2 (0.8)	0.997
Uses antacids	5 (33.3)	25 (41.7)	0.400	16 (25.4)	68 (27.3)	0.636
Ate raw fruits or vegetables in the last 2 weeks	11 (73.3)	56 (93.3)	0.036	38 (60.3)	147 (59.0)	0.860
Family unable to reheat food in the last week	9 (60.0)	29 (48.3)	0.332	25 (39.7)	59 (23.7)	0.004
Consumed food or beverage prepared outside the household in the last week	13 (86.7)	60 (100.0)	0.999	43 (68.3)	211 (84.7)	0.002
Family gets water to drink from an improved water source	13 (86.7)	56 (93.3)	0.999	44 (69.8)	161 (64.7)	0.053
Frequency of treating drinking water^c^						
Almost never	4 (26.7)	8 (13.3)	0.235	8 (12.7)	19 (7.6)	0.538
Sometimes	8 (53.3)	32 (53.3)		22 (34.9)	91(36.5)	
Often	1 (6.7)	7 (11.7)		8 (12.7)	31 (12.4)	
Almost always	1 (6.7)	0 (0.0)		3 (4.8)	14 (5.6)	
Always	1 (6.7)	13 (21.7)		22 (34.9)	94(37.8)	
Frequency of handwashing with soap and water (times per day)^c^						
Not at all	5 (33.3)	1 (1.7)	0.015	1 (1.6)	6 (2.4)	0.434
Once	4 (26.7)	24 (40.0)		17 (27.0)	54 (21.7)	
Twice	5 (33.3)	26 (43.3)		30 (47.6)	117 (47.0)	
Three times	1 (6.7)	9 (15.0)		13 (20.6)	57 (22.9)	
Four times or more	0 (0.0)	0 (0.0)		2 (3.2)	15 (6.0)	
Able to observe toilet facility	3 (20.0)	11 (18.3)	0.755	35 (55.6)	107 (43.0)	0.028
Improved toilet system^d^	2 (66.7)	8 (72.7)	0.999	29 (82.9)	83 (77.6)	0.648
Number of households that share the toilet	2.00 [1.50, 3.00]	2.00 [1.00, 2.00]	0.999	2.00 [1.00, 3.00]	1.00 [1.00, 2.00]	0.020

a Continuous variables are presented as Median (interquartile range) and proportions are presented as Number (%) unless stated otherwise. n is as listed in the table header, unless stated otherwise.

b Univariable conditional logistic regression adjusted for matching factors (age, gender). *P*-values not reported for matching factors.

c Ordinal variables modeled as continuous variables.

d Only if able to observe toilet. n = 3 and 11 for cases and controls in the vaccine effectiveness study, respectively. n = 35 and 107 for cases and controls in the bias-indicator study, respectively.

In the VE case-control study, 53.4% of cases reported any vaccination compared to 71.7% of controls (Table 2). Among those vaccinated, 50.0% of cases received two doses compared to 72.1% of controls. In univariable analyses adjusted for matching factors, vaccination with two doses of OCV was associated with a reduction in the risk of cholera (crude relative risk [RR] 0.23, 95% CI 0.05-1.12), corresponding to VE of 77.0% (95% CI –12.0-95.0%). In the VE case-control study, attending school was the only variable associated with both cholera and vaccination at a significance level of *P* < 0.20 in univariable analyses. In multivariable analyses adjusting for matching factors and attending school, the adjusted RR of cholera among those receiving two doses of OCV was 0.31 (95% CI 0.06-1.71), corresponding to adjusted VE of 69.0% (95% CI –71.0-94.0%). Corresponding crude and adjusted VE estimates for one dose of OCV and for either one or two doses of OCV are shown in Table 2. In a sensitivity analysis using a culture-based definition of cholera, where only those cases that were positive for *V. cholerae* by culture were included as cases, the adjusted VE of two doses of Euvichol® was 84.0% (95% CI –106.0-99.0%) (Supplementary Table 2).

**Table 2:** Euvichol® vaccine effectiveness in a case-control study in Haiti, 2018-2020^a^.

VE case-control	Cases (N = 15)	Controls (N = 60)	Crude RR (95% CI)^b^	Crude VE (95% CI)^b^	Adjusted RR (95% CI)^c^	Adjusted VE (95% CI)^c^
**Number of doses**						
Two	4 (26.7%)	31 (51.7%)	0.23 (0.05-1.12)	77% (-12 - 95%)	0.31 (0.06 - 1.71)	69% (-71 - 94%)
One	4 (26.7%)	12 (20.0%)	0.78 (0.17 - 3.66)	22% (-266 - 83%)	1.9 (0.28 - 12.4)	-90% (-1140 - 72%)
None	7 (46.7%)	17 (28.3%)	Ref	Ref	Ref	Ref
**Vaccination card available**^d^	0/8 (0%)	6/43 (14.0%)				

Bias-indicator case-control	Cases (N = 63)	Controls (N = 249)	Crude RR (95% CI)^b^	Crude VE (95% CI)^b^	Adjusted RR (95% CI)^e^	Adjusted VE (95% CI)^e^
**Number of doses**						
Two	17 (27.0%)	86 (34.5%)	0.62 (0.30 - 1.26)	38% (-26 - 70%)	-	-
One	12 (19.0%)	43 (17.3%)	0.95 (0.44 - 2.05)	5% (-105 - 56%)	-	-
None	34 (54.0%)	120 (48.2%)	Ref	Ref	-	-
**Vaccination card available**^d^	1/29 (3.4%)	10/129 (7.8%)				

CI, confidence interval; RR, relative risk; Ref, reference; VE, vaccine effectiveness.

a Doses of vaccines are by self-report.

b Adjusted for matching factors (age and gender).

c Adjusted for matching factors (age and gender) and whether the participant attended school.

d Number of individuals who provided a vaccination card for review among individuals who reported receiving the vaccine.

e Adjusted analyses were not performed given that no factors were associated with both cholera and vaccination at our prespecified threshold.

In the bias-indicator case-control study, 46.0% of cases reported vaccination compared to 51.8% of controls (Table 2). No variables were associated with both cholera and vaccination at a significance level of *P* < 0.20 in univariable analyses. The crude VE of two doses of OCV was 38.0% (95% CI –26.0-70.0%) (Table 2).

## Discussion

In this study, we demonstrate a two-dose Euvichol® OCV VE of 69% between 10 and 27 months following a mass OCV campaign implemented as part of comprehensive cholera control efforts in Haiti. This level of protection is in line with previous studies of the Shanchol™ OCV in Haiti and elsewhere, suggesting that two doses of the Euvichol® OCV confer protection against cholera in this setting.

To our knowledge, this is only the second study evaluating the effectiveness of Euvichol®. A 2018 case-control study evaluated the effectiveness of Euvichol-Plus® immediately following a cholera outbreak in Lusaka Zambia, demonstrating an OCV effectiveness of 81% [24]. That study enrolled cases retrospectively and was subject to selection bias as only a small subset of culture-positive cases (28.5%) had sufficient information to allow follow-up. Additionally, only culture was used to define cholera. The lower sensitivity of culture compared to PCR as a diagnostic for the clinical disease of cholera in patients with acute watery diarrhea has been increasingly documented [19,22,23], and PCR has been increasingly used in studies of cholera VE [15], [16], [17], [18]. Our study builds on this evidence by providing an estimate of OCV effectiveness from a prospective study with an accompanying bias-indicator assessment, and by using a more sensitive diagnostic methodology in the form of RT-PCR. When we defined cases of cholera as only those cases of acute watery diarrhea that were positive by culture, we estimated VE of 84%, similar to findings from Zambia. This possible difference between VE estimates raises important questions regarding the impact of diagnostic test sensitivity and specificity on oral cholera VE estimates. Given its increased sensitivity, PCR diagnostics may lead to more complete case ascertainment, which could lead to more valid estimates of VE.

Our findings are in line with previous estimates of whole-cell killed OCV effectiveness (predominantly reflecting the effectiveness of Shanchol™) including in Haiti where we found a two-dose VE of 62% (95% CI 6.0-85%) within 22 months and 76% (95% CI 59.0-86.0%) within 4 years [12,25]. More broadly, a systematic review and meta-analysis from 2017 summarized a mean VE of 76% [6]. These data on the field effectiveness of Euvichol® come at a critical time for global cholera control efforts as Euvichol-Plus® becomes the sole globally available OCV and the supply of OCV remains unable to keep up with demand [1,26]. Although further studies and evaluations of Euvichol® vaccine campaigns are needed, our findings suggest that the field effectiveness of Euvichol® is similar to Shanchol™.

Critical questions regarding the effectiveness of OCV remain. Namely, data on long-term VE remains sparse. One-dose VE data is also limited. In this study, the sample size was too small to evaluate single-dose VE with accuracy. Additionally, VE in key groups including children and immunocompromised hosts, and the effectiveness of vaccination in the setting of different transmission scenarios (outbreak, epidemic, and endemic settings) requires further study.

Findings from this study should be interpreted within the context of the following limitations: First, the overall number of enrolled cases in the study was low, resulting in wide CIs, and limiting our assessment of VE in subgroups of interest. The low case numbers likely resulted from both the campaign and other interventions and activities to control cholera in Haiti, and the waning phase of the national epidemic itself at the time. Additionally, we were delayed in immediately starting the study due to the emergent nature of the campaign and institutional review board delays. Despite this, point estimates are in line with previous estimates of OCV effectiveness. Second, vaccination status, as with many other OCV effectiveness studies, was assessed by self-report, and rates of vaccine card availability were low [18,27,28]. Ascertaining vaccination status by self-reporting can result in recall bias. We employed several strategies such as clarifying questions and pictures of OCV, discussed in the methods, to improve the validity of self-reported vaccination status, and we believe that OCV recall in this setting is high given that this is the only vaccine in this region administered to adults as part of mass vaccination campaigns (the national Expanded Immunization Program only distributes vaccine for children under the age of 3). Additionally, although the rate of available vaccine cards was low, in a previous study, we estimated OCV effectiveness using both self-reported vaccination and card-confirmed vaccination and noted no major differences in 2-dose VE [12,29]. Finally, to address overall bias in this study, we conducted a bias-indicator study, which demonstrated a lower effectiveness of OCV when protecting against non-cholera diarrhea. This association was substantially attenuated as compared to the primary VE case-control study. This could suggest that our main VE estimate is over-estimated; however, consistency with previous estimates substantiates the validity of these findings.

In conclusion, in this case-control study, we demonstrate that the Euvichol® OCV has an estimated two-dose VE of 69% at 2 years following a vaccine campaign in central Haiti, in line with previous estimates of VE of whole-cell killed OCVs. Further studies are indicated in different outbreak and epidemiologic settings to evaluate effectiveness over the long term and among subgroups including children. As formulations of Euvichol® become the major supply of OCV globally and cholera remains a global concern, further evaluations to guide the implementation of this OCV as part of multi-sectoral, comprehensive cholera control and prevention efforts will be critical to diminish the global burden of cholera.

## Declarations of competing interest

The authors have no competing interests to declare.

## Appendix Supplementary materials

Image, application 1
