# Supplementary material for: Effectiveness of the Euvichol® oral cholera vaccine at 2 years: A case-control and bias-indicator study in Haiti
Source: Int J Infect Dis. 2024 Feb;139:153–8. doi: 10.1016/j.ijid.2023.11.022 (PMC10784151; doi:10.1016/j.ijid.2023.11.022)
Supplement: Supplementary file 1 [file mmc1.docx]

**Supplementary Materials: Effectiveness of the Euvichol^®^ oral cholera vaccine at 2 years: a case-control and bias-indicator study in Haiti**

Wilfredo R. Matias,^1,2,3^ Yodeline Guillaume,^3^ Gertrude Cene Augustin,^4^ Kenia Vissieres,^4^ Ralph Ternier,^4^ Damien M. Slater,^1, 5^ Jason B. Harris,^1,5^ Molly F. Franke,^6^ Louise C. Ivers^1,3,6,7^

^1^Division of Infectious Diseases, Massachusetts General Hospital, Boston, MA, USA

^2^Division of Infectious Diseases, Brigham and Women’s Hospital, Boston, MA, USA

^3^Center for Global Health, Massachusetts General Hospital, Boston, MA, USA

^4^Zanmi Lasante, Croix-des-Bouquets, Haiti

^5^Department of Pediatrics, Harvard Medical School, Boston, MA, USA

^6^Department of Global Health and Social Medicine, Harvard Medical School, Boston, MA, USA

^7^Harvard Global Health Institute, Cambridge, MA, USA

Corresponding author:

Wilfredo R. Matias, MD MPH

Massachusetts General Hospital

Division of Infectious Diseases

55 Fruit St, BUL-130, Boston, MA, 02114, USA

+1-617-726-3812

wmatias@bwh.harvard.edu

ORCID: 0000-0002-5371-1354

**Table of contents**

1. Title Page
2. Table of Contents
3. Description of the RT-PCR assay for *V. cholerae* used in the study
4. Supplementary Table 1. Clinical presentation and treatment of diarrheal disease cases in a case-control study in Haiti, 2018 – 2020
5. Supplementary Table 2. Euvichol® vaccine effectiveness in a case-control study in Haiti, using a culture-based definition of cholera, 2018 – 2020.

**Description of the RT-PCR assay for *V. cholerae* used in the study**

We used a TaqMan Real-Time polymerase chain reaction (RT-PCR) to the *ctxA* gene. A 6-mm diameter hole punch was used to sample filter cards, cleaning with sterile water and flame-sterilizing with ethanol between each card. Three punches were taken for DNA extraction using the DNAeasy PowerLyzer PowerSoil kit (Qiagen, Germantown, MD), following manufacturer’s instructions. The *ctxA* gene RT-PCR consisted of the following oligonucleotides (F, 5’-CATAGAGCTTGGAGGGAAGAGC-‘3, R, 5’-TCGTCAAGGAATTTTACACCTAGACT-3’, P, 56-FAM/GGGAATGCT/ZEN/CCAAGATCATCGAT GAGTAATACTTG/3IABkFQ). Oligonucleotides were obtained from Integrated DNA Technologies (Coralville, IA). Final primer and probe concentrations for all sets were 200 nM. Reactions were prepared using 5 microliters of template in a 20 microliter PCR using 2x iQ Multiplex Powermix (Bio-Rad, Hercules, CA). Positive and negative controls were included on each run. The PCR conditions used were a single 2-minutes 95°C hot-start activation step, followed by 40 cycles of 95°C for 10 seconds, and 60°C for 1 minute, using an ABI 7500 Fast instrument (Thermo Fisher Scientific, Waltham, MA).

| **Supplementary Table 1. Clinical presentation and treatment of diarrheal disease cases in a case-control study in Haiti, 2018 – 2020^a^** | | |
| --- | --- | --- |
|  | Cholera diarrhea cases  (n = 15) | Non-cholera diarrhea cases  (n = 63) |
| *Vibrio cholerae* O1 stool culture positive | 8 (53.3%) | - |
| Serotype Inaba | 8 (100.0%) | - |
| *V. cholerae* cholera toxin RT-PCR cycle threshold (median [IQR]) | 21.96 (19.58 – 24.68) | - |
|  | Available Clinical Data^b^ Cholera diarrhea cases(n = 15) | Available Clinical Data^b^ Non-cholera diarrhea cases (n = 32) |
| Time from symptom onset to admission (days) | 1.00 [0.00, 1.00] | 1.00 [0.00, 1.00] |
| Oral rehydration solution (ORS) taken at home | 4 (26.7%) | 12 (37.5%) |
| Dehydration stage at presentation |  |  |
| None or mild | 4 (26.7%) | 13 (40.6%) |
| Moderate | 6 (40.0%) | 15 (46.9%) |
| Severe | 5 (33.3%) | 4 (12.5%) |
| ORS administered | 15 (100.0%) | 32 (100%) |
| Intravenous fluids (IVF) administered | 12 (80.0%) | 16 (50.0%) |
| Volume of IVF administered (Liters)^c^ | 7.5 [3.50, 8.00] | 4.00 [2.00, 5.00] |
| Antibiotic prescribed | 11 (73.3%) | 25 (78.1%) |
| If yes, which antibiotic? |  |  |
| Doxycycline | 11 (100.0%) | 18 (72.0%) |
| Erythromycin | 0 (0.0%) | 7 (28.0%) |
| Other | 0 (0.0%) | 0 (0.0%) |
| Admitted overnight | 15 (100.0%) | 29 (90.6%) |
| Disposition after treatment |  |  |
| Discharged | 14 (93.3%) | 31 (96.9%) |
| Transferred | 0 (0.0%) | 0 (0.0%) |
| Died | 0 (0.0%) | 0 (0.0%) |
| Abandoned treatment | 1 (6.7%) | 1 (3.1%) |
| ^a^Continuous variables are presented as median (IQR) and proportions are presented as Number (%) unless stated otherwise. | | |
| ^b^Clinical data were missing for 31 of the 63 non-cholera diarrhea cases. | |  |
| ^c^Among those administered intravenous fluids (12 cholera diarrhea cases and 16 non-cholera diarrhea cases) | | |
| ^d^Other includes amoxicillin, ciprofloxacin, azithromycin, or another antibiotic | |  |

| **Supplementary Table 2. Euvichol^®^ vaccine effectiveness in a case-control study in Haiti, using a culture-based definition of cholera, 2018 - 2020^a^** | | | | | | |
| --- | --- | --- | --- | --- | --- | --- |
| **Vaccine effectiveness case-control** | **Cases**  **(N = 8)** | **Controls**  **(N = 32)** | **Crude RR**  **(95% CI)^b^** | **Crude VE**  **(95% CI)^b^** | **Adjusted RR**  **(95% CI)^c^** | **Adjusted VE**  **(95% CI)^c^** |
| **Number of doses** |  |  |  |  |  |  |
| Two | 2 (25.0%) | 17 (53.1%) | 0.17 (0.02 - 1.57) | 83% (-57 - 98%) | 0.16 (0.01 - 2.06) | 84% (-106 - 99%) |
| One | 1 (12.5%) | 5 (15.6%) | 0.58 (0.05 - 6.20) | 42% (-520 - 95%) | 0.99 (0.07 - 13.4) | 1% (-1240 - 93%) |
| None | 5 (62.5%) | 10 (31.3%) | Ref | Ref | Ref | Ref |
| **Vaccination card available^d^** | 0/3 (0%) | 4/22 (18.2%) |  |  |  |  |
| **Bias-indicator case-control^e^** | **Cases**  **(N = 69)** | **Controls**  **(N = 273)** | **Crude RR^b^**  **(95% CI)^b^** | **Crude VE**  **(95% CI)^b^** | **Adjusted RR**  **(95% CI)^f^** | **Adjusted VE**  **(95% CI)^f^** |
| **Number of doses** |  |  |  |  |  |  |
| Two | 19 (27.5%) | 99 (36.3%) | 0.57 (0.29 - 1.14) | 43% (-14 - 71%) | - | - |
| One | 14 (20.3%) | 50 (18.2%) | 0.90 (0.43 - 1.88) | 10% (-88 - 57%) | - | - |
| None | 36 (52.2%) | 124 (45.4%) | Ref | Ref |  |  |
| **Vaccination card available^d^** | 1/33 (3.0%) | 12/149 (8.1%) |  |  |  |  |
| ^a^Doses of vaccine are by self-report | | | | | | |
| ^b^Adjusted for matching factors (age and gender) | | | | | | |
| ^c^Adjusted for matching factors (age and gender) and whether the participant attended school. | | | | | | |
| ^d^Number of individuals that provided a vaccination card for review among individuals that reported receiving vaccine. | | | | | | |
| ^e^2 cases were missing culture data, so these were removed with their 8 matching controls | | | | | | |
| ^f^Adjusted analyses were not performed given that no factors were associated with both cholera and vaccination at our prespecified threshold. | | | | | | |
